# Diversity Models and Applications of 3D Breast Tumor-on-a-Chip

**DOI:** 10.3390/mi12070814

**Published:** 2021-07-12

**Authors:** Kena Song, Xiangyang Zu, Zhe Du, Zhigang Hu, Jingjing Wang, Jinghua Li

**Affiliations:** College of Medical Technology and Engineering, Henan University of Science and Technology, Luoyang 471023, China; kenasong@haust.edu.cn (K.S.); zu.xiangyang@163.com (X.Z.); duzhe@haust.edu.cn (Z.D.); hu.robert@163.com (Z.H.); 9906134@haust.edu.cn (J.W.)

**Keywords:** breast tumor-on-a-chip, breast models, applications

## Abstract

Breast disease is one of the critical diseases that plague females, as is known, breast cancer has high mortality, despite significant pathophysiological progress during the past few years. Novel diagnostic and therapeutic approaches are needed to break the stalemate. An organ-on-chip approach is considered due to its ability to repeat the real conditions found in the body on microfluidic chips, offsetting the shortcomings of traditional 2D culture and animal tests. In recent years, the organ-on-chip approach has shown diversity, recreating the structure and functional units of the real organs/tissues. The applications were also developed rapidly from the laboratory to the industrialized market. This review focuses on breast tumor-on-a-chip approaches concerning the diversity models and applications. The models are summarized and categorized by typical biological reconstitution, considering the design and fabrication of the various breast models. The breast tumor-on-a-chip approach is a typical representative of organ chips, which are one of the precedents in the market. The applications are roughly divided into two categories: fundamental mechanism research and biological medicine. Finally, we discuss the prospect and deficiencies of the emerging technology. It has excellent prospects in all of the application fields, however there exist some deficiencies for promotion, such as the stability of the structure and function, and uniformity for quantity production.

## 1. Introduction

Breast disease is a serious factor threatening women’s health, such as hyperplasia, fibroadenosis, fibroadenomas, benign tumor and carcinoma. In the diversity of diseases, breast cancer is one of the primary causes of death for women worldwide. Nearly 2.1 million cases of breast cancer occur every year globally, and about 0.62 million die from breast cancer [1]. Furthermore, breast diseases are not exclusive to women since male breast diseases were reported. Therefore, many questions need to be answered, including the pathogenic mechanisms, drug metabolic pathways, finding the effective targets and so on.

Traditional models were based on the 2D culture in Petri dish and animal testing. However, 2D culture differs greatly from the real situation in vivo caused by the absence of the microenvironment. In contrast, animal testing is more reliable, however, it is hard to ignore the difference in microenvironments between human and animals. Recently, artificial organs were proposed for building a closer model to humans. Two strategies are being rapidly developed, one of which is organoid culture techniques, and the other the organ-on-chip based on microfluidic technology. The former relies on three-dimensional (3D) culture of mammalian stem cells with sequential addition of growth factors. However, it is difficult to control conditions precisely for reproducing the complex and dynamic microenvironment. By contrast, organ-on-chip could regulate the microenvironment accurately based on microfluidic chip. Researchers are forming alliances the organoids culture techniques with microfluidics platform to address the limitations of organoids culture technique, and also improve the authenticity of the organ-on-chip. In recent years, the artificial organs based on microfluidics present diversity, including multiple types of organs such as breast, lung, live, heart, kidney, and so on, and even for the same organ diverse models are being created.

The breast tumor-on-a-chip is a typical representative of the organ chips. Lots of breast models on chip have been created in decades. To reproduce the more realistic breast structure and function, stem cells or human primary cells were utilized as the derivation to fabricate spheroid, luminal, or other functional units through differentiate, self-organization, or 3D bioprinting. Based on pathological characteristics, double or triad organs were fabricated on microfluidic chip to build the breast cancer metastasis models, such as breast-live-on-chip, breast-vessel-on-chip, breast-heart-on-chip. The applications were concentrated in mechanism studies or drug screening. In this view, we will provide a detailed discussion on the diversity models and application of breast tumor-on-a-chip in recent decades, with a special focus on the structures, fabrications, approaches, and the applications in biological medicine.

## 2. The Physiology and Pathology of the Breast

**Figure 1:**
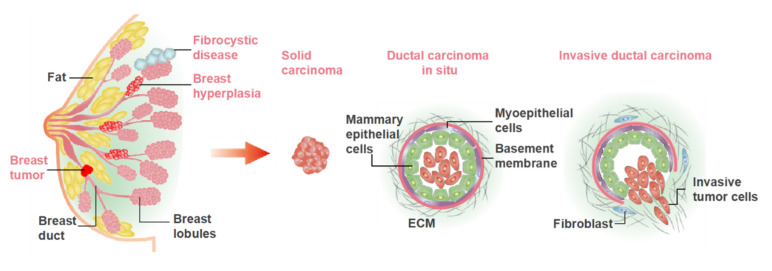
The physiological structure of mammary gland and the common breast diseases. The benign diseases mainly include breast hyperplasia, fibrocystic disease, benign tumor, etc. The malignant tumors mainly include solid carcinoma and ductal carcinoma.

The human breast is mainly composed of glands, ducts, adipose tissue and fibrous tissue, the internal structure of which is like a tree growing upside down. The physiological structure is shown in Figure 1. The breast gland is composed of 15–20 gland lobes, while each gland lobe is divided into several gland lobules, and each glandular lobule is composed of 10–100 acini. These acini are closely arranged around the small ducts, and the opening of the acinar is connected with the small ducts. Multiple small breast ducts merge into the interlobular milk duct, and the multiple interlobular breast ducts further merge into a whole glandular breast duct, also known as the lactiferous duct. There are 15–20 lactiferous ducts in human breast. The lactiferous duct is narrow at the nipple, and then enlarged into the ampulla, called the lactiferous sinus, which has the function of storing milk.

Breast diseases are derived from breast glands, fat, lymph, blood vessels, nipples and other breast-related tissues, including breast inflammatory diseases, benign breast lesions, breast malignant tumors, congenital abnormalities, and male breast development. The common breast diseases are illustrated in Figure 1. Bacterial/viral infections and the cellular carcinogenesis are two mainly causes of breast disease. At present, the treatment principle of breast disease is to remove the cause, treat symptomatically, reduce recurrence, and improve survival rate and quality of life after treatment. Many females lose breasts due to breast disease, especially breast cancer. Therefore, it remains important to fabricate the novel models for research and therapeutic approaches to breast diseases.

## 3. Various In Vitro Breast Models on a Microfluidic Chip

### 3.1. Spheroid Model

Since the spheroid model was introduced into cancer research in 1971 [2], it has been favored by researchers and has become a conventional in vitro model for tumor research. A spheroid is aggregated by multicellular tumor cells and retains the capacities of proliferation and invasion, the features of which are similar to a solid tumor in a body. In a spheroid, the states of cells are quite different from shallow to deep location caused hypoxia. The surface cells possess active proliferative capacity, called the proliferating cell area, while the middle cells are in a dormant state, called the resting cell area, and the cells of the central area are apoptosis due to the severely deficit of oxygen and nutrient, called the necrotic cell area [3]. That is similar to a small breast tumor nodule without blood vessels in the body. However, the cancer microenvironment could be vascularized in the body to gain oxygen and nutrition. For this reason, researchers attempted to explore tumor angiogenesis in a spheroid model to mimic the vascularized cancer microenvironment [4,5]. In breast tumor-on-a-chip, the microfluidic chip could be not only the application site of a previous spheroid, but also integrate the preparation process. The distribution is flexible by arrays or out-of-order in the microfluidic chip. Diverse methods of spheroid preparation have been developed in recent years, such as the liquid overlay method, pendant drop method, and bioprinting. The size of the spheroid could be steerable by controlling the seeding numbers, bioprinting scale and culture periods [6,7,8,9,10,11]. The designs of a spheroid are diverse according to requirements. We summarize and categorize the diverse designs as below.

#### 3.1.1. Homotypic Spheroid

**Figure 2:**
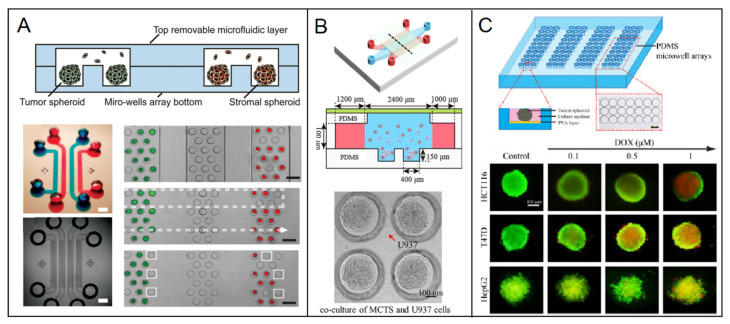
Diverse spheroids such as the breast models in a microfluidic chip. (**A**) Different cell spheroids of uniformly sized breast tumor cell of MCF-7 and fibroblast were prepared and systematic analyzed in a microfluidic device [12]. Open access. (**B**) Monocytes are co-cultured with low invasive low invasive T47D spheroids or high invasive MD-MBA-231 spheroids in a collagen matrix-embedded microfluidic device [17]. Reprinted with permission from ref. [17]. Copyright 2018 Chinese Academy of Medical Sciences. (**C**) A great number of uniform 3D tumor spheroids of breast cancer cell T47D were formed as well as other human cancer cell lines of HepG2 and HCT116 in a microfluidic chip for drug susceptibility testing [19]. Reprinted with permission from ref. [19]. Copyright 2015 Elsevier B.V.

Homotypic spheroid is a conventional model for 3D culture because it is convenient to fabricate. Breast cancer cell lines are commonly used to construct spheroid, including high invasive MDA-MB-231, BT20, low invasive MCF-7, Hs578, Hcc1937 and noninvasive MCF-10A. Liang Zhao et al. [12] aggregated breast tumor cells of MCF-7 and fibroblast cells, respectively, to prepare diverse homotypic spheroids in a top-removable microfluidic device and analyze different spheroids on a single device (Figure 2A). Hildegonda P.H. Naber et al. [13] generated homotypic MCF-10A spheroid cell cultures embedded in a 3D collagen matrix in vitro to study TGF-β-induced invasion. Cancer stroma and the extracellular matrix (ECM) are the important factors for cancer progress, including few fibroblasts, mesenchymal cells and collagen, elastin, fibrin, fibronectin, respectively [14,15,16]. Therefore, stroma cells and ECM were considered by neighboring them or embedding into the spheroid respectively to enrich the microenvironment in in vitro models. Tianying Yuan et al. [17] developed a microfluidic chip-based 3D breast cancer model by co-culturing monodisperse breast tumor spheroids aggregated by T47D or MDA-MB-231 with monocytes in a 3D collagen matrix to investigate macrophages and ECM effect on tumor cell migration (Figure 2B). Personalization is a developing trend in biomedicine. Primary cells are the best choice to include in the drug-delivery system and models. Manuela Cipolletti et al. [18] exploited a novel anti-estrogen discovery platform using primary breast cancer cells to identify new Food and Drug Administration (FDA)-approved drugs inhibiting E2:ER signaling to cell proliferation. Yongli Chen et al. [19] developed a microwell array microfluidic system to form diverse spheroids of breast cancer cell T47D and other human cancer cell lines of HepG2 and HCT116 for drug susceptibility testing and offline drug signaling pathways. The response of doxorubicin and paclitaxel on different types of spheroids were simultaneously performed in the system (Figure 2C). Although the microenvironment is partly ignored inside a homotypic spheroid, this could be remedied by modifying the environment adjacent to the spheroid. Therefore, it is still a common model for cancer research, and it provides a basis for other researches due to its simple preparation, such as imaging technology [20], dynamic self-assembly [21], intratumoral microvessels [22], and drug delivery [23]. Also due to the controllable and high efficiency in preparation, homotypic spheroid has excellent potential to promote industrial applications in the future.

#### 3.1.2. Heterotypic Spheroid

In order to simulate the tumor tissue in the body more realistically, the heterotypic spheroid has been developed. Firstly, stromal cells were aggregated with tumor cells, including fibroblasts, endothelial cells, and adipocyte cells, considering the factors of the microenvironment. Fibroblast plays a pivotal role in cancer progression and resistance to anti-cancer treatments [24,25]. It has been proved it interacts with secret hepatocyte, fibroblast, epithelial and tumor mass, which are involved in the proliferation of cancer cells and the activation of the apoptosis resistance [25,26,27]. Epithelial cells could be induced to mesenchymal transition by invasive malignant cells. It could construct intratumoral microvessels in solid tumors. Adipocytes play a critical role in the stiffness of the tissue microenvironment, which influences the cancer process, including proliferation, differentiation and migration. Marco P. Carvalho et al. [28] obtained a heterotypic spheroid as the realistic model of breast tumors through co-culturing MCF-7 and human dermal fibroblasts with the ratio of 3:1 and 1:1. Madhuri Dey et al. [4] developed a physiologically relevant 3D vascularized breast cancer micro-environment comprising of metastatic MDA-MB-231 cells and human umbilical vein endothelial cells. C. C Yang et al. [29] combined heterocellular tumor spheroids with breast tumor cells and adipocyte cells to investigate the behavior of breast cancer cells in response to different environment stimuli in 3D microenvironment.

Secondly, the immune system is the most complete defence of the body to incursions, including cancerous cells, bacteria, virus. Therefore, the heterotypic spheroid embedding immune cells, such as monocyte, macrophages, and star cells, is the dominant modality to study breast tumors. Aereas Aung et al. [30] developed a multicellular tumor-on-a-chip platform by breast cancer cells, monocytes, and endothelial cells to examine the effect of cancer cell-monocyte interaction on T-cell recruitment. Moreover, the cancer cells with different phenotypes were co-cultured to be heterotypic spheroids, which are used to mimic the cell subpopulations with varying degree of invasiveness in primary tumors. Siddarth Chandrasekaran et al. [31] generated a spheroid model that included breast tumorigenic breast cell lines BT20, MCF-7 and a non-tumorigenic epithelial cell line MCF-10A to mimic tumor heterogeneity in vitro.

The development of a complex heterotypic spheroid is a trend aiming to mimic the real situation in the body. However, there are fewer cell types and components used to construct tumor spheroid models currently due to the difficulty of multicellular co-culture. Therefore, to overcome the predicament of complex co-culture and stability of multi-heterotypic spheroid still needs further research [32,33].

### 3.2. Lumen Models

Mammary ducts are tubular structures to produce or transport milk. However, the ductal dysfunction in breasts are early hallmarks of multiple human diseases, such as the tumorigenic process. The pathology typically occurs in lobules and terminal ducts of mammary glands. Ductal breast cancer typically progresses from a benign lesion to invasive ductal carcinoma to ultimately metastasize. In the metastasizing process, intravasation and extravasation occur through blood vessels, in which the tubular structure plays an important role. Microfluidic lumen-based systems are microscale models that recapitulate the anatomy and physiology of mammary ducts and blood vessels. The lumen generation mainly relies on the self-organization feature of epithelial cells. Cell-patterning and hydrogel molding are the main methods of generating a luminal structure in microdevices. The endothelial cells always were seeded onto a 2D surface within a microchannel, such as a porous membrane or hydrogel interface. However, gravity is a hindrance for constructing the ceiling and profile of a complete lumen. Overturning the devices is a general approach to overcome gravity. As an example, Deepika Devadas et al. [34] placed the devices upside down and right side up for 15 min each to ensure uniform seeding along lumen walls, ceiling, and floor.

#### 3.2.1. Breast Ductal Model

Several microfluidic approaches in 2D were developed to mimic the local monolayer epithelial cell sheet structure. [35,36] This could mimic the local functional unit, however a 2D cell sheet model could not recapitulate the ductal geometry structure and physicochemical cues presenting in the breast tumor environment. Recently, the emergence of organotypic microfluidic models recapitulated the 3D mammary ductal structure and integrated components of the tumor microenvironment.

**Figure 3:**
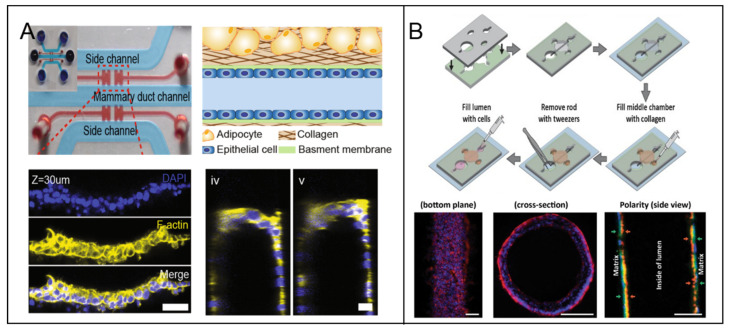
The lumen models in breast tumor-on-a-chip. (**A**) A stable hemi-channel mammary duct was formed on the spatially modified COL1 hydrogel under optimized coating condition. [37] Open access. (**B**) A mammary duct was fabricated on an extracellular matrix (ECM) lumen hollow, which was fabricated by presetting and pulling out of a polydimethylsiloxane (PDMS) rod [41]. Open access.

Hemi-channel self-assembled by epithelial cells is adopted by some researchers as the ductal model. Youngkyu Cho et al. [37] recapitulated the lumen structure to mimic the morphology and function of mammary epithelium in a 3D microfluidic platform. ECM based hydrogels were served as scaffolds to offer biophysical features to facilitate the construction of various epithelial types (Figure 3A). Meggie M.G. Grafton et al. [38] developed a hemi-channel U-shaped luminal structure in microfluidic chip to mimic the tree-like ductal system of breast. Using this model, basoapical polarity was observed when they co-cultured phenotypically normal and diseased cells and tumor nodules in the hemi-channels epithelial cells structure. That is consistent with the counterpart in vivo, however is not observed in traditional 2D platform. Pierre-Alexandre Vidi et al. [39] cultured a normal breast luminal epithelium on semicircular acrylic support to mimic mammary ducts. They found that the morphologies of the cells from tumor nodules were distinct from their counterparts cultured on flat surfaces. Further, tumor nodules in the hemi-channels of the breast tumor-on-a-chip were significantly less sensitive to anticancer drug, e.g., bleomycin and doxorubicin, compared to their 2D monolayer counterparts. That proved breast tumor geometry affected the biological characteristics.

The complete channel comprised by epithelial cells was fabricated successfully as the breast ductal model. Jonathan Kulwatno et al. [40] modeled an organotypic mammary duct as a channel in a collagen matrix and lined it with basement membrane. The distinct characteristics of hyperplasia and invasion of breast cancer progression were observed in the model. Furthermore, the distinct behaviors of different cell types were displayed in the luminal model, as an example normal mammary epithelial cells MCF10A formed a single-cell layer on the lumen surface, whereas MCF10CA1 aggregated several cell layers thick. Deepika Devadas et al. [34] presented two parallel 3D luminal structures using mammary epithelial cells and vascular endothelial cells, respectively, in a microfluidic co-culture system, to mimic mammary duct and neighboring vessel. For higher reproducibility of luminal dimension, ECM hydrogels with patterned lumen were used as the framework to generate a physiologically relevant model of the mammary duct. Molly M. Morgan et al. [41] placed polydimethylsiloxane (PDMS) rods into the device chamber before pouring hydrogel into it. When the hydrogel was cured, a hollow lumen structure was remained after the PDMS rod being pulled out. MCF-7 solution was pipetted into the hollow ECM lumen, followed by incubation and flipping from bottom to top every 20 min for 100 min. An organotypic mammary model was generated consisting of a collagen-embedded duct structure (Figure 3B).

#### 3.2.2. Breast-Vasculature Interaction Model

Vasculature is an essential component in the complex process of cancer progression. To metastasize, breast tumor cells break away from primary carcinoma intravasate into the vasculature, and extravasate to distant organs to form secondary tumors. Therefore, the vasculature has a significant role in metastasizing process. The tumor-associated vasculatures include intratumoral and neighboring microvessels, which are quite different from the normal ones in structure and function. The endothelial cells of the vasculatures are disorganized or absent in endothelium. Furthermore, the vasculatures are highly responsive to angiogenic growth factors and hyperpermeable to blood plasma and plasma proteins. The features above provide convenience to tumor cells metastasizing. Microfluidic breast-vascular models came into being to provide a new platform to mimic the realistic situation in vivo [42]. Quantity microfluidic lumen-based models have been established to recapitulate the process of intravasation, extravasation and tumor angiogenesis in cancer metastatic cascade.

Andrew D. Wong et al. [43] developed an artificial vessel embedded in an extracellular matrix to study the intravasation process of breast cancer cell MDA-MB-231. The 3D lumen vessel was generated by nitinal rod templates and subsequently lined with human dermal microvascular endothelial cells. In the model, dual-labeled MDA-MB-231 breast cancer cells were embedded within the collagen matrix as either single cells or clusters. Invasion and intravasation of breast cancer cells were captured through time-lapse imaging in the matrix surrounding the blood vessels. Mouhita Humayun et al. [44] employed a human organotypic breast cancer cell extravasation model consisting of a tubular endothelial vessel generated from induced pluripotent stem cell derived endothelial cells within a collagen-fibrinogen matrix. Breast cells were cultured in the lumen of vessel to study extravasation in the cancer–vascular interactions. The system identified that cancer-vascular crosstalk increased the levels of secreted IL-6, IL-8, and MMP-3. Venktesh S. Shirure et al. [45] created a quiescent perfused 3D microvascular network prior to loading tumor cells to mimic biological mass transport near the arterial end of a capillary in the tumor microenvironment. The platform provided the opportunity to simultaneously and dynamically observe hallmark features of tumor progression including cell proliferation, angiogenesis, cell migration and tumor cell intravasation.

The lumen model is one of the models with most potential to develop in vitro, because it a common structure in body, such as glands, acinus, vessels. However, the preparation has had a low effect because of the high dependence on self-organization and scaffold. Therefore, it is an arduous task to improve the preparation efficiency for industrial promotion.

### 3.3. Designable Patterned Co-Culture Models

A microfluidic chip provides a convenient way to fabricate the pattern structure of a breast tumor. Diverse patterns were formed according to requirement, such as patterned co-culture, tissue engineering of biomimetic structure, homotypic and heterotypic cell–cell interaction, and cell–matrix interaction. Traditionally, the co-culture models were generated by seeding two or more types cells on a substrate randomly. However, they are limited by the inability to vary local cell seeding density and the degree of cell–cell contact. To overcome the limitations, micropatterned co-culture based on microfluidic chip were employed to regulate cell-cell and cell-matrix interactions by enhancing spatial localization of cells, ECM and biological factors [46].

The patterned co-cultures were conventionally generated relying on supporting materials or scaffold structure, such as selective adhesion substrates, soft lithography-based patterning, and switchable surface-based patterning. To obtain the selective adhesion of micropatterned substrates, adhesion molecules were coated on substrates with micropatterns, such as integrins and cadherins. Either cells were cultured on substrates with selective adhesion, or different cell types exhibited different levels of adhesiveness against various surfaces. That enabled localizing specific cell types to micropatterned regions on the substrate in order to obtain micropatterns [46,47].

**Figure 4:**
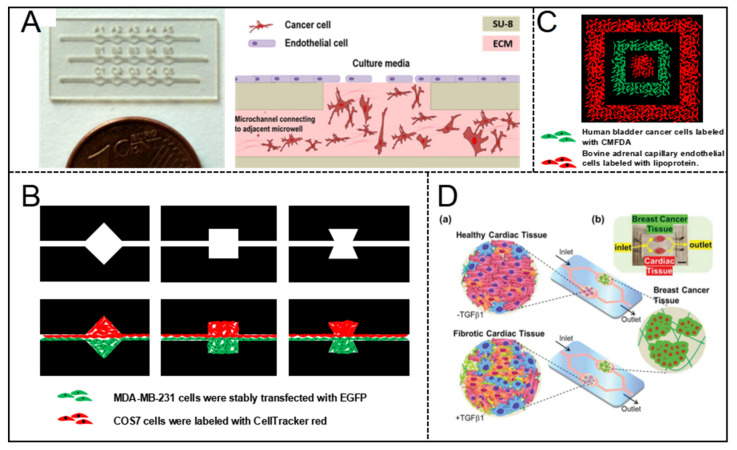
Designable patterned co-culture model in breast tumor-on-a-chip. (**A**) The co-culture of a 3D breast tumor model and a 2D endothelium model in a multiwell capillarity-based microfluidic device [48]. Open access. (**B**) Patterned spheroids of various geometries and compositions were formed to manipulate cell-cell interaction dynamics [49]. Adapted from [49], Data from [49]. (**C**) A concentric square was patterned by two cell types and proteins in a 3D microfluidic system [50]. Adapted from [50], Data from [50]. (**D**) A dual-organ platform of breast and heart were fabricated in microfluidic chip to investigate the chemotherapy-induced cardiotoxicity [51]. Open access.

María Virumbrales-Muñoz et al. [48] reported a microfluidic device for co-culture of a 3D breast tumor model and a 2D endothelium model for cross-talk and drug-delivery studies. Several linear arrays of microwells were fabricated by photolithography firstly. The 3D tumor model was created by embedding breast cancer cells MDA-MB-231 in a 3D collagen matrix, on the top of which human umbilical vein endothelial cells (HUVEC) monolayers were prepared as 2D mimics of the endothelial barrier. The patterned co-culture models were fabricated by the device with generating gradients of oxygen and cell preferential proliferation (Figure 4A). The patterned model is anticipated to facilitate drug screening in a relevant microenvironment.

Yu-suke Torisawa et al. [49] described a sandwich microfluidic with a semi-porous membrane to form patterned co-culture model. Arbitrary cellular arrangements were enabled by regulating the geometric features of the bottom channel and flow hydrodynamics. Patterned co-culture models with various geometries and compositions were fabricated successfully (Figure 4B).

Daniel T. Chiu et al. [50] fabricated a concentric square pattern of two cell types by using a 3D PDMS stamp (Figure 4C). Before patterning cells, the stamp was preset with coating bovine serum albumin (BSA) for preventing cell attachment. Suspensions of cells were introduced into the channels and incubated. The patterned model was established after the stamp was removed.

### 3.4. Breast–Other Organ Interaction Model

Complex physiological pathways exist in disease progress and drug metabolites, as well as organ interactions. The single organ model is unable to recapitulate the complexity, functional changes and integrity of organ function despite their advanced capabilities. Therefore, multi-organ-on-a-chip is a future trend to meet the requirement of mechanism research and biological medicine through integrating multiple organ units on a microfluidic chip. It enables a new strategy to research cancer because it could recapitulate all steps of the metastatic cascade of cancer, including epithelial-mesenchymal transition (EMT), invasion, intravasation, extravasation, metastasis. Also, the platform makes it possible to regulate the microenvironment accurately and establish high-throughput drug screening by microfluidic techniques [52,53].

A multiorgan microphysiological system was developed by integrating lung, liver, and breast cancer on a microfluidic chip to study the delivery pathways of curcumin. Three cell lines of A549, HepG2 C3A, MDA-MB-231were used to create the organs of lung, liver, and breast cancer in three chambers of the microfluidic unit, respectively. The liver cells and tumor cells were plated on 3D scaffold separately, then placed into their compartment respectively. Utilizing the multiorgan microphysiological system, the authors found intravenous delivery decreased breast cancer viability significantly compared to inhalation therapy [54].

Cardiotoxicity is one of the most serious side effects of cancer chemotherapy and, as an example, breast cancer patients with preexisting cardiac dysfunctions may experience different incident levels of chemotherapy-induced cardiotoxicity (CIC). For investigating the early signs of CIC, Junmin Lee et al. [51] built a dual-organ platform of heart-breast cancer based on microfluidic chip (Figure 4D). A cured PDMS containing dual-organ geometry was used consisting of three different domains for locating cardiac tissues, breast cancer tissues, and fluidic respectively. Cardiac spheroids derived from human pluripotent stem cells were encapsulated into gelatin methacryloyl (GelMA) hydrogels, combining the key cell types of cardiomyocytes, fibroblasts, myofibroblasts in the heart. Breast cancer spheroids were prepared and encapsulated in hydrogels to generate the breast cancer tissue model. The production trend of biomarkers evaluated by using the platform matched the outcomes from conventional enzyme-linked immunosorbent assays well.

The breast–other organ interaction model is a prototype body-on-chip. Undoubtedly, it has an excellent development prospect. However, it is still at the stage of the concept and an extremely simple model, distant from industrial promotion. Therefore, to promote the complexity and the stability of the multi-organ chip approaching the real body is a future direction for engineering the body-on-chip.

### 3.5. The 3D Extracellular Matrix in Breast Tumor-on-a-Chip

#### 3.5.1. Matrix Candidate

The ECM is an important component in a 3D microenvironment of cells in vivo. Accumulating evidence has revealed that ECM is critical to maintain cell activities and functions, including proliferation, migration, apoptosis, and responses to drugs, etc. The existence of the ECM makes the in vitro models realistic both in structure and function. Hakkinen, et al. reported that the migration rate of fibroblasts was higher in the 3D matrix of collagen or cell-derived matrix compared to the counterpart in a 2D environment [55]. Drug-response studies in a 3D environment are necessary due to the differ existing in surface area to volume ratio between 3D cell cultures and the 2D cell monolayers [56]. Furthermore, the integrins and cadherins on the cell membrane have been proved to sense the chemical composition and physical properties of the ECM by mechanism studies [57]. Therefore, recreating the 3D matrix in a microfluidic chip is an important aim of organ-on-chip engineering.

To simulate the features of the realistic matrix, the characteristics of porous and permeability is paid attention to. Hydrogels and electrospun fibers are the two families match the requirements in the engineering of organ-on-a-chip. Traditionally, the alternative hydrogels mainly originate from animals or plants, called natural hydrogels, such as collagen, matrigel. However, the available hydrogels show variance due to the rapid development in the biochemical characteristics and biocompatibility of artificial hydrogels, including synthetic and hybrid materials. The natural hydrogels occupy a natural advantage through the feature of cytocompatible and native cell-binding ligands, and could be used to replicate the real component and ratio of the matrix in the body [58,59]. However, they are limited by low controllability due to the natural stability of the molecules [60]. Synthetic hydrogels are chemically synthesized from precursor molecules to customize the desired mechanical properties with high reproducible. For enhancing the cell adherence, the chemical modifications always are added to the molecules [61,62]. Hybrid hydrogels are synthesized by mixing the different types of bio-sourced small molecules to complement each other’s shortcomings [63].

Electrospun fibers were utilized as the scaffolding materials for 3D cell culture due to its tunable diameter and mechanical stiffness, and the ability to embed particles/compounds. Both synthetic and natural polymers could be implemented in electrospinning. Polycaprolactone (PCL) and polylactic acid (PLA) are the two common materials used to prepare the electrospun fibers due to their biocompatibility and biodegradable nature [64,65,66]. To generate more physiologically relevant ECMs, the natural polymers were explored for electrospinning, such as the solutions of collagen and fibronectin [67]. Silk fibroin has gained attention for the application as electrospun fibers recently due to its remarkable biocompatibility, high water and oxygen uptake, and tunable mechanical properties [68,69]. Importantly, electrospun fibers enjoy numerous factors to be potential candidates of biosensors in microfluidic chips, including the high surface to volume ratio, analogous scale to bioactive molecules, relatively defect-free properties. That provides the benefit to diagnostic devices by integrating the biosensing processes into a miniaturized microfluidic chip.

#### 3.5.2. Hypoxia Model

Different oxygen levels exist in a solid tumor in body. It conventionally exhibits hypoxia in the central part of the solid tumor. The hypoxia conditions are proved to promote tumor progression and being resistance to therapy. It is essential to replicate complex oxygen levels in in vitro models. Therefore, hypoxia models were developed to match the special environment of a tumor. It is a challenge to develop the approaches of controlling the oxygen level precisely in models in vitro. A microfluidic chip provides the basis for accurately controlling fluids and gases, and could offer advanced spatio-temporal control of physico-chemical parameters. Many approaches are utilized to generate the hypoxia condition in breast tumor-on-a-chip, including physical, chemical and biological methodologies.

A spheroid has the geometrical property similar to solid tumor in vivo, providing the native properties of gradient oxygen situation [70]. For the study of hypoxia in tumor spheroids, Ilana Berger Fridman et al. [71] developed a multi-layer microfluidic device to generate 3D breast tumor spheroids under the microenvironment of five levels of oxygen concentrations with high throughput. The author exploited the oxygen gradient model to investigate how two chemotherapeutic drugs, Doxorubicin and Tirapazamine, affect breast tumor spheroids.

**Figure 5:**
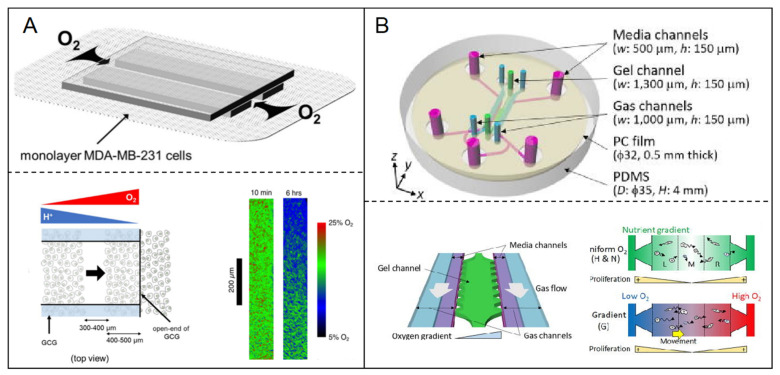
Hypoxia model in breast tumor-on-a-chip. (**A**) The gradient of oxygen concentration was established in microfluidic chip by cellular oxygen consumption in the relatively closed spaces [71]. Open access; (**B**) A linear oxygen gradient from 3% to 17% was generated through the oxygen permeability of ECM. The response of breast cancer cells to different oxygen tension was investigated using the hypoxia microfluidic system [72]. Open access.

Homoplastically, metabolic gradient was manufactured through nutrition gradient supported. The gradient of oxygen concentration was established by cellular oxygen consumption in the relatively closed spaces. Eiji Takahashi et al. [72] proposed a simple microfluidic system to produce the gradients of energy substrates and metabolites, including H^+^, oxygen in monolayer-cultured cells (Figure 5A). Using this system, the authors monitored the migratory behaviors of MDA-MB-231 breast cancer cells in the gradient conditions of pH and/or oxygen.

The oxygen permeability of the ECM was utilized to generate hypoxia. Rei Koens et al. [73] developed a double-layer microfluidic device including two parallel gas channels located above the media, separated by a gel channel to enhance oxygen exchange. A gas-impermeable polycarbonate film was embedded above the channels to prevent the diffusion of atmospheric oxygen (Figure 5B). The device could generate a linear oxygen gradient from 3% to 17% across the gel channel. Under the oxygen gradient condition in the device, the authors investigated the response of breast cancer cells to different oxygen tension.

Oxygen-scavenging chemicals could be utilized to generate the hypoxia condition. Venktesh S. Shirure et al. [74] lowered the oxygen concentrations through sodium sulfite reacting with oxygen in a PDMS-based tissue culture system. A flexible theoretical framework was used to provide fine spatial and temporal control of oxygen tension in the microfluidic system. The authors investigated the impact of hypoxia inducible factor-1α on the properties of MDA-MB-231 breast cancer cells using this hypoxia microfluidic system.

The breast models on a microfluidic chip are diverse, as shown above. There are advantages for each model, however, deficiencies also exist in the models. We summary the advantages and deficiencies of each model in Table 1.

## 4. Manufacturing of Artificial Breast Microfluidic Devices

PDMS is the most popular material to manufacture microfluidic devices in the lab. However, it is limited by expense for industrialization. Therefore, several materials were developed allowing rapid-prototyping and low-cost for microfluidic applications. The process technologies were broadened from soft-lithography to thermoplastics and laser-cut. The diverse materials and manufacture technology strategies are displayed in Table 2.

## 5. Application of In Vitro 3D Breast Tumor-on-a-Chip

The artificial breast on chip has been widely used in biomedical science and technology due to its functional responses at the level of organ or tissue. It has prospective application prospects caused by the replacement of the animal test as well as improving the discovery processes of new drugs for personal healthcare. It is being developed rapidly from disease models in the lab to personalized medicine. So far, the main applications focus on pharmaceutical development and the mechanism of investigation of breast cancer. Other applications were also explored in recent years.

### 5.1. Pharmaceutical Benefits

The organ-on-chip has revolutionized the drug screening process from animal models to personalized medicine. The application covers the scopes of efficiency and toxicity of drugs, the pharmacokinetics/pharmacodynamics, administration, distribution, metabolism and excretion. That is a benefit for ecological protection, personal healthcare and economic effects.

The resistance and heterogeneous response are the significant challenges of anti-cancer drug development and threaten the prognosis of cancer patients. A typical chemotherapeutic drug doxorubicin (DOX) targeting breast cancer was evaluated by a breast tumor-on-a-chip in which three types of human breast cancer cell (MCF-7, MDA-MB-231, and SUM-159PT) were co-cultured within a three-dimensional extracellular matrix perfused with interstitial fluid. The authors displayed the diverse characteristics and reported that a higher drug resistance and cell type dependence in the artificial organ platform compared to the traditional 2D culture [90].

Henriette L. Lanz et al. [91] focused on the therapy selection of breast cancer. For conquering the poor predictors and long period of the traditional methods, they investigated the feasibility of a high-throughput organ-on-chip platform to select therapies. The triple negative breast cancer cell lines MDA-MB-231, MDA-MB-453, and HCC1937 were selected to embed into extracellular matrix comprised by Matrigel, BRCA1, or collagen. A series of anti-cancer drugs (paclitaxel, olaparib, cisplatin) were evaluated by exposing cells to them. The microfluidic organ platform allowed the simultaneous culture of 96 perfused micro tissues, enabling drug screening of patient-derived material with high throughput.

In some cases, the drug resistance is caused by tumor vasculature, which could crease a hostile tumor microenvironment to nourish cancers. For studying the tumor responses to the anti-cancer drugs incorporating the effects of blood flow, Yuji Nashimoto et al. [92] presented a tumor-on-a-chip platform with a tumor vascular network, enabling the evaluation of tumor activities with intraluminal flow. A vascular network was constructed through inducing angiogenic sprouts by fibroblasts in the tumor spheroid. In contrast to the results under static conditions, the drug administration under perfusion conditions in this platform did not show the dose-dependent effects of anticancer drugs on tumor activities.

To get closer to personalized treatment, a detailed protocol was developed to incorporate the functional vascularized tissues and organoid technology. Two or more tissues were connected easily through a common vasculature, which also played a recruitment role to attract non-lical immune cells as a protective measure. Breast tissue was aggregated into the organ-on-chip with other organ tissues (i.e., hepatic, cardiac, patient pancreatic organoid) and ECM (i.e., fibrinogen, matrigel, and other appropriate hydrogels). That is an alternative method for personalized drug screening or uncovering subtle biological responses [93]. Otherwise, primary cells were seeded into the organ-on-chip for personalized medicine. Bone metastases occur in advanced breast cancer patients by approximately 70% through a close interaction between breast cancer circulating tumor cells and the local bone marrow cells including pericytes and osteoblasts. For developing the precision medicine of the bone metastases from breast cancer, Vera Mayo et al. [94] isolated human primary cells of mesenchymal stem/stromal cells according to the CD146 presence to recapitulate the endosteal niche. Several candidate drugs were evaluated in pre-clinical to clinical trials using this platform. That provided a support for the engineering of an organ-on-chip approach and personalized medicine development.

### 5.2. Biological Mechanism Studies

The exploration of a path mechanism is the unavoidable way to become aware of the disease and expose treatment targets. The artificial organ-on-chip also contributes to the mechanism studies by providing the platform for recapitulating the disease and microenvironment in vitro. That is beneficial for conditional regulation and data acquisition compared with animal models.

To study the effect of the acidic microenvironment on breast tumor viability, Sandra F. Lam et al. [95] developed a bifurcated microfluidic device supporting two different microenvironments to compare them directly. The breast tumor model was comprised by triple-negative breast cancer cell MDA-MB-231 and fibroblasts in the microfluidic device. The breast model in one of the two chambers was treated by acid-neutralizing CaCO3 nanoparticles. Using the platform, the authors indicated that the acidic microenvironment could stimulate cancer cell reprograming to suppress tumor growth and invasion.

The early metastatic niche (EMN) is a transient microenvironment which reflect the complex competitive mechanisms of pro- and anti-metastatic stimuli to cancer cells in the progression. The transient microenvironment is complexed by the crosstalk among cancer cells, platelets, leukocytes and endothelial cells. It could increase the cancer cells’ ability to extravasate and colonize a new nidus. To better understand the complex crosstalk, Martina Crippa et al. [96] developed a human “EMN-on-a-chip” by creasing a microenvironment situation around breast cancer tissue, which is more similar to the counterpart in vivo. They showed that the transendothelial migration of cancer cells was significantly increased when exposed in neutrophils and platelets compared to cancer cell alone. Furthermore, the EMN-on-a-chip was exploited in combination with multi-culture experiments. The authors showed that the transition markers of epithelial to mesenchymal cancer cells could be increased to express by platelets stimuli.

In cancer progression, the T-cell recruitment is a significate immunization strategy, which is determined by the complex interaction between cellular and tumor microenvironments. To clear the mechanism of T-cell recruitment, Aereas Aung et al. [30] developed a perfusable multicellular tumor-on-a-chip including breast cancer cells, monocytes, endothelial cells, and a gelatin hydrogel ECM. The authors found that the aggregated cells in a spheroid with hypoxia microenvironment could recruit more T cells compare to the dispersed cancer cells. Furthermore, the chemokine secretion is a significant factor affecting the T-cell recruitment.

### 5.3. Industrial Progress of Breast Tumor-on-a-Chip

In recent decades, the organ-on-chip has been progressing rapidly from concept to application. It has been budding in worldwide markets, such as biomedical, drug-delivery, immunoassay and other biotechnological applications. The application market is favored by many well-known pharmaceutical and biotechnological companies, such as Abbott, Ibidi. Small and medium-sized enterprises also scramble to enter the field, such as Elvesys, Dolomite, Fluigent. That demonstrates the excellent application prospects of organ-on-chip.

In breast disease research, the microfluidic devices are being exploited and popularized for the early detection and diagnosis of the disease and drug screening. BioIVT’s Elevating Science Industry (USA) developed a device characteristics of oncology tissue microarrays as a screening tool for multiple cancer patient tissue samples. The device combined the function of immunohistochemistry and in situ hybridization in the oncology tissue microarrays, enabling discovery of new proteins and genetic markers for disease diagnosis and validation. The high throughput is one of the maximum advantages of the microfluidic platform, as well as less consumption. Mimetas^TM^ (Layton, the Netherlands) developed an organplate^®^ platform with high-throughput to fabricate the breast model, embedding breast cancer cells and tissue into 3D hydrogel. In the microfluidic platform, 96 microtissues could be cultured instantaneously by perfusing limited consumption of culture medium or growth factors. That allows the full advantage of limited patient-derived materials to drug screening. OrganoPlate^®^ Graft is a platform developed by Mimetas^TM^ supporting personalized medicine of appropriate drug selection and therapy. The platform permits spheroids, organoids and tumors to develop vasculature in a microfluidic chip, allowing the combination of primary cells culture and drug administration for personalized medicine.

Other products are also launching in succession with extensive application scopes. InSphero (USA) developed a customized tumor model enabling it to support the drug toxicity assessment of diversity organs, including lung, kidney, liver, pancreas, ovary and breast cancers. The model was developed by optimizing tissue structure and the biological characteristics of primary tumors, which is beneficial to personalized medicine. Ibidi (Martinred, Germany) manufactured a series device for studying cancer behavior, metastasis and chemotaxis. Emulate^TM^ (Boston, MA, USA) is also working on the development of microfluidic chip for diverse applications, including understanding diseases complexity, drug development, and personalized medicine.

We summarize typical companies with the explored models in breast tumor-on-a-chip systems and the specific applications in Table 3.

## 6. Summary and Future Perspectives

Diverse breast models have been developed for the breast tumor-on-a-chip in recent years. In this review, the models are summarized and categorized by the characteristic of structure and function. The integrated factors are increasingly complex, from homotypic spheroid to multiple organs, with complicated microenvironments.

The simple functional units were duplicated, firstly, such as homotypic spheroid. That was followed rapidly by a heterotypic spheroid or other co-culture models, which integrated the mesenchymal cells and immune system, or other adjacent tissues. Relatively, a homotypic spheroid is too monotone to the real body, however, it is convenient for industrial promotion due to the high preparation efficiency. The heterotypic spheroid has an excellent perspective because it could recapitulate the complexity of tumors in the body. However, the co-cultivation dilemma is an obstacle for complex model developed rapidly, which needs more research to overcome. The characteristic structures of the breast were incorporated along with technical progress, such as the ductal and luminal structure, as well as the function based on the special structures. That is a perspective model to organs in vitro, because lumen exists in many organs in body, including gland, acinus, vessel, etc. However, the preparation efficiency and stability are limited by the dependence on self-organization, which needs to be promoted. The lesion and the corresponding complex 3D microenvironment, including the ECM, adjacent blood vessel and other organs, were also recapitulated tentatively in the breast tumor-on-a-chip, which is similar to the body-on-chip. However, the complex models are just a prototype for the body-on-chip although it is an excellent trend in development. Recently, the applications of breast tumor-on-a-chip have mainly concentrated in the fields of biomedicine and biological mechanism study, although the organ-on-chip was being applied in radiobiology. Industrial applications of breast tumor-on-a-chip have also been initially developed, however, the models are mainly based on simple tissue microarray or cell aggregation without a complex microenvironment.

In the future, the breast tumor-on-a-chip has an excellent development prospects whether in industrial applications or fundamental mechanism studies. Biological medicine still has the greatest application prospects, especially in personalized medicine. Along with the complexity and maturation of the organ models, animal testing in the traditional pharmaceutical procedures will be replaced by the organ-on-chip. Personalized medicine will be a vigorously developed and promoted direction in the future. However, there are multiple dilemmas that need to be overcome, as shown above. At present, only a few generations of model have reached the point of promotion, while many models are still in progress at the laboratory stage, far from reaching the stability of industrial standards. Therefore, more research and effort are needed to achieve industrial promotion.

## Figures and Tables

**Table 1:** The advantages and deficiencies of the breast models in breast tumor-on-a-chip.

Model Type		Advantages	Deficiencies	Reference
**spheroid model**	Homotypic spheroid model	simple, diversified preparation technology, easy to prepare, efficient, controllable	monotony, very different from the real situation in body	[75,76,77]
	Heterotypic spheroid model	simulate the tumor tissue in the body more realistically	co-cultivation dilemma, low efficiency	
**lumen model**	Breast ductal model	enable simulation of breast duct structure and function	dependence on the scaffold and self-organization, low efficiency	[42,78,79]
	Breast-vasculature model	enable simulation of the infiltration and extravasation of the cancer invasion		
**other patterned models**		designable	high dependence on the scaffold, low efficiency	[46,49]
**breast-other organ model**		complex, diverse, simulates the interaction between tissues and organs in vitro, close to in vitro body model	co-cultivation dilemma, low efficiency	[51,52,53]
**hypoxia model**		simulates the hypoxia microenvironment around the tumor in body, diversified methods, controllable	difficult to prepare, severely affected by airtightness and matrix permeability	[74,80,81]

**Table 2:** The available materials of microfluidic chips in the engineering of an organ-on-chip.

Material	Manufacture Technology	Structure Features of the Microfluidic Chip	Reference
PDMS	soft-lithography, CO_2_ laser-cut	serpentine micro-channels	[82]
Poly-Methyl-MethAcrylate (PMMA)	CO_2_ laser-cut	integration of electrospun membranes into laser-structured PMMA modules	[83]
Mylar	CO_2_ laser-cut	a storage cavity embedded in a microchannel	[84]
Poly-lactic acid (PLA)	thermoplastic	multilayer structure with microchannels	[85]
Poly-Carbonate (PC)	thermoplastic	a PC membrane sandwiched between two COP layers in a multilayered device	[86]
PolyStyrene (PS)	thermoplastic	assembled flexible compartment structure	[87]
Cyclic Olefin Polymer (COP)	thermoplastic	Array of straight microchannels	[88]
Cyclic Olefin Copolymer (COC)	thermoplastic	a sealed structure with channels and the inlet and outlet manifold	[89]

**Table 3:** The pharmaceutical and biotechnological companies and the explored breast tumor-on-a-chip systems.

Company	Chip Type or Model Characteristics	Specific Applications
BioIVT’s Elevating Science Industry (USA)	Tissue Microarrays	screening multiple cancer patient tissue samples
Mimetas™ (The Netherlands)	High-throughput OrganoPlate^®^ platform to culture breast cancer cells and tissue embedded in a 3D hydrogel.	drug screening of patient-derived materials; personalized medicine for appropriate drug selection and therapy.
InSphero (USA)	3D InSight™ Microtissues model; a customized tumor model of monoculture or co-culture model based on a 3D Select™ system	in vitro drug testing; drug toxicity assessment
μFluidics (USA)	Microfluidic point-of-care chips	rapid detection of biomarkers from various diseases
Emulate™ (USA)	Organ chips	understanding diseases complexity; drug development and working towards the development of personalized medicine
Luohua (China)	Tumor Organoid Chip	tumor precision diagnosis and treatment
Scienion (Germany)	Array-based multi-parameter detection	multi-parametric miRNA analysis from a variety of cancer (breast, lung, thyroid, pancreatic, and liver)
IDEX Health and Science (USA)	Integrating a silicon nanowire-based biosensor on organ chip	detecting tumor biomarkers (protein and circulatory tumor DNA) from liquid biopsies.
Ibidi (Germany)	A variety of microdevices based on microfluidic chip	study cancer cells‘ behavior, metastasis and chemotaxis
